# Checkmite!? Is the Resistance to Phytophagous Mites on Short and Stocky Wild *Oryza* Species?

**DOI:** 10.3389/fpls.2018.00321

**Published:** 2018-03-13

**Authors:** Raul A. Sperotto, Giseli Buffon, Joséli Schwambach, Felipe K. Ricachenevsky

**Affiliations:** ^1^Graduate Program in Biotechnology, University of Taquari Valley, Univates, Lajeado, Brazil; ^2^Biological Sciences and Health Center, University of Taquari Valley, Univates, Lajeado, Brazil; ^3^Graduate Program in Biotechnology, University of Caxias do Sul, Caxias do Sul, Brazil; ^4^Graduate Program in Agrobiology, Federal University of Santa Maria, Santa Maria, Brazil; ^5^Graduate Program in Cell and Molecular Biology, Federal University of Rio Grande do Sul, Porto Alegre, Brazil

**Keywords:** gibberellin, jasmonate, mite resistance, plant defense, wild species

Plants must effectively defend against environmental stresses to survive in nature. However, immunity to disease is costly and often comes with a significant growth inhibition and yield penalty (Yang et al., 2012; Huot et al., 2014; Huang et al., 2017; Ning et al., 2017). Hormones play important roles in regulating plant growth and stress responses (Heinrich et al., 2013). Gibberellins (GAs) and jasmonates (JAs) are two types of essential phytohormones that control many aspects of plant growth and development in response to environmental and endogenous signals. GA regulates many essential plant developmental processes (including stem and leaf elongation), while JA plays a dominant role in mediating plant defense, especially to herbivores attack (Hou et al., 2013). Even though GA and JA antagonize each other in regulating plant growth and defense response via interaction between JAZs and DELLAs proteins (De Bruyne et al., 2014; Song et al., 2014; Chaiwanon et al., 2016), the exactly way how plants coordinate the fluctuating growth-defense dynamics is not well understood. Especially, the role of GA in growth-defense conflicts during herbivory is yet to be characterized. Several works have been addressing the plant dilemma between “to grow” and “to defend” in response to various stimuli, clearly indicating that plants need to prioritize GA- or JA-induced responses. Heinrich et al. (2013) showed that high levels of JA antagonize the biosynthesis of GA and inhibit the growth of *Nicotiana attenuata* stems. In rice, GA application was found to decrease resistance to the hemibiotrophic rice pathogens *Magnaporthe oryzae* (Mo) and *Xanthomonas oryzae* pv. *oryzae* (Xoo) (Yang et al., 2008; Qin et al., 2013). The GA biosynthetic pathway and signaling cascade were shown to be regulated by JA during Mo and Xoo interactions with rice plants. It was also shown that the only DELLA protein in rice, SLR1, is crucial to integrate GA and JA (as well as salicylic acid) crosstalk (De Vleesschauwer et al., 2016). In agreement with that, rice plants overexpressing a GA deactivating enzyme accumulated low levels of GA and displayed enhanced resistance to Mo and Xoo, whereas plants harboring loss-of-function mutations in the same gene were more vulnerable to these pathogens (Yang et al., 2008; De Bruyne et al., 2014).

Upon attack by the chewing herbivore *Chilo suppressalis*, rice plants activate the expression of OsWRKY70, a transcription factor that physically interacts with W-box motifs and prioritizes defense over growth by positively regulating JA and negatively regulating GA biosynthesis (Li et al., 2015). Two groups investigated the growth/defense response of rice plants infested by brown planthopper (BPH) insect: (1) Wang et al. (2015) detected a shift from growth to defense in response to BPH infestation, evidenced by down-regulation of GA-related genes, decreased GA levels, increased JA levels, and reduced plant growth; (2) Qi et al. (2016) showed that plants over-expressing *OsJMT1* (JA carboxyl methyltransferase, which is up-regulated by BPH infestation and is a key enzyme in methyl-JA biosynthesis pathway) exhibited increased MeJA levels and reduced height. In line with these findings, we previously detected lower expression of three GA biosynthetic pathway-related genes (*OsGA2ox1, OsGA2ox3*, and *OsGA20ox1*) in rice leaves infested with the phytophagous mite *Schizotetranychus oryzae*, when compared with control leaves. The expression of *OsAOS* (allene oxide synthase), which catalyzes the committed step in JA biosynthesis, was only detected in infested leaves (Buffon et al, 2016). Altogether, these results suggest that JA-related responses antagonize the biosynthesis of GA and GA-related responses during herbivory.

Wild plant species have been widely recognized as valuable source of resistance genes for developing herbivore-resistant cultivars. For example, *Oryza brachyantha* is resistant to the rice leaf folder *Cnaphalocrocis medinalis* (Ramachandran and Khan, 1991; Ricachenevsky et al., 2018). To date, 10 QTLs and one causative gene have been identified from six wild rice species (*O. officinalis, O. eichingeri, O. minuta, O. latifolia, O. rufipogon*, and *O. australiensis*—Huang et al., 2013; Zhang et al., 2014; Hu et al., 2015; Ji et al., 2016). With this in mind, we asked ourselves whether wild rice cultivars could also present some degree of resistance to *Schizotetranychus oryzae* mite infestation. Surprisingly, the wild rice genotypes tested (*O. glaberrima* and *O. barthii*) were characterized as highly sensitive to *S. oryzae* infestation, being even more sensitive than cultivated *O. sativa* genotypes (Figures 1A,B). Similar results were reported by Veasey et al. (2008), which tested the infestation of *S. oryzae* in four wild rice species (*O. glumaepatula, O. latifolia, O. alta*, and *O. grandiglumis*), and Chandrasena et al. (2016), which tested the infestation of panicle rice mite *Steneotarsonemus spinki* in five wild rice species (*O. nivara, O. eichingeri, O. rufipogon, O. granulata*, and *O. rhizomatis*), with no signs of mite resistance.

**Figure 1:**
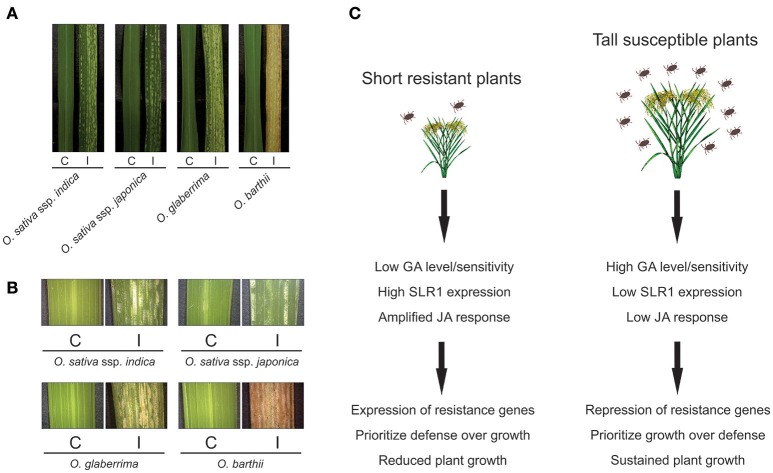
Visual characteristics of leaves from *O. sativa* ssp. *indica, O. sativa* ssp. *japonica, O. glaberrima*, and *O. barthii* plants, under control (C) and infested (I) conditions **(A)**. Detailed view of these leaves under stereomicroscope **(B)**. Schematic model of the different responses probably employed by short resistant and tall susceptible plants **(C)**.

Taking into account the antagonism between JA and GA, and that breeding of cultivated rice during the Green Revolution has selected for low GA/GA-insensitive genotypes, as shown by the semi-dwarf phenotype of modern rice cultivars (Spielmeyer et al., 2002), we believe the mite-sensitivity presented by the wild rice species could be explained, at least partially, by a presumable high GA:JA ratio in these plants. Tested wild species are tall plants, varying from 1.5 to 5 m, and probably have high levels of GA synthesis. It is important to highlight that a plant's height is not only dependent on the GA level, and resistance to herbivores is not only dependent on the JA level. However, considering the high variability found on the genomes of wild rice species and the high number of already identified insect-resistance genes, we hypothesize that a short (or a semi-dwarf) wild rice species could present a significant level of mite-resistance. In line with our assumption, the semi-dwarf IR36 rice cultivar (one of many of the Green Revolution which replaced many local strains and genetic diversity previously found in rice paddies, resulted from a cross-breeding of IR8 with 13 parent varieties from six nations and a wild species of rice, *O. nivara*) is resistant to many pests and diseases, including green leafhopper (*Nephotettix virescens*), BPH, stem borer (*Chilo* sp.), blast blight (*Pyricularia oryzae*), bacterial blight (*Xanthomonas campestris* pv *oryzae*), tungro, and grassy stunt viruses (Innes, 1992).

Therefore, we would like to suggest short *Oryza* species and genotypes as primary sources of herbivory tolerance, including mites. We should expect low GA levels/sensitivity, and therefore high SLR1 (the sole DELLA protein in rice) levels in these plants. Accumulated SLR1 will amplify the JA-response, driving plant resources toward defense instead of growth (Chaiwanon et al., 2016; De Vleesschauwer et al., 2016). Obviously, we do not expect the GA-JA switch to be the sole determinant of resistance. However, plants with high GA stimulus are more likely to have lower levels of SLR1, freeing JAZ proteins to sequester JA-response activation transcription factors and in turn attenuate JA-mediated resistance (Figure 1C). Thus, we should focus our efforts in searching for useful genes in genotypes that are already primed to JA defense responses (i.e., plants with low GA level/sensitivity and therefore high SLR1 levels, which would amplify JA-responses and drive plant resources toward defense instead of growth).

We suggest *O. minuta, O. meyeriana, O. neocaledonica*, and *O. schlechteri* (http://www.knowledgebank.irri.org/images/docs/wild-rice-taxonomy.pdf) as possible sources of mite-resistance genes. *O. minuta* (2*n* = 48, BBCC genome, 1 m tall, perennial) exhibits significant potential to resist to several pests/diseases (http://archive.gramene.org/species/oryza_species/o_minuta.html), including blast blight, bacterial blight (BB), white-backed planthopper (WBPH), and brown planthopper (BPH) (Amante-Bordeos et al., 1992; You et al., 2007; Rahman et al., 2009; Asaf et al., 2016, 2017), which are damaging to the growth and yield of cultivated rice. Few studies have been conducted to identify and transfer the resistance genes from *O. minuta* to cultivated rice (Amante-Bordeos et al., 1992; Rahman et al., 2009). However, no hybrid with commercial rice cultivar with elevated resistance to herbivores have been developed so far. *O. meyeriana* (2*n* = 24, GG genome, about 50 cm tall, perennial) is adapted to survive in harsh environments and possesses many useful traits absent in cultivated rice, including high resistance to rice blast and bacterial blight, which has been transferred to cultivated rice (*O. sativa*) (Yan et al., 2004; Han et al., 2014; He et al., 2015; Chen et al., 2016; Cheng et al., 2016). *O. neocaledonica* (2*n* = 24, GG genome, 60–80 cm tall, perennial), is the latest species described in the genus *Oryza* (Nayar, 2014). First considered a subspecies/variety/population of *O. meyeriana* (Vaughan, 2003), it was later recognized as a valid *Oryza* species (Clayton et al., 2010 - https://www.kew.org/data/grasses-db.html). Surprisingly, no studies are known to have been done on *O. neocaledonica* (Nayar, 2014), evidencing an unexplored gene diversity. *O. schlechteri* (2*n* = 48, HHKK genome, 30–90 cm tall, annual), found in undisturbed forests, is the least studied species in the genus (Brar and Singh, 2011). Based on bioclimatic analysis, Atwell et al. (2014) pointed *O. schlechteri* as a candidate species for flooding tolerance. Regarding biotic stress response, no studies have been done with this species, and for this reason is an irreplaceable material for improving the cultivated varieties.

Even though herbivore resistance is commonly a genetically determined trait that shows heritable genetic variation (Muola et al., 2010), it is important to highlight that before including a plant material as a primary source in a breeding program, is essential to know the heritability of the trait and how stable the trait would be when transmitted to the offspring. Therefore, it would be interesting to examine whether and how environmental factors regulate GA:JA ratio/crosstalk and mite resistance in these short and stocky wild rice species. Also, we are aware that crossing often fails to generate fertile hybrids between cultivated rice and wild species because of reproductive barriers (Han et al., 2014). However, the use of asymmetric somatic hybridization has proved to be effective (Jena, 2010). Therefore, searching for mite resistance genes taking GA-JA crosstalk into account, together with comprehensive screens in the wild rice diversity, should be fruitful to develop mite resistance in susceptible cultivated rice lines.

## Author contributions

All authors listed have made a substantial, direct and intellectual contribution to the work, and approved it for publication.

### Conflict of interest statement

The authors declare that the research was conducted in the absence of any commercial or financial relationships that could be construed as a potential conflict of interest. The reviewer MAHR and handling Editor declared their shared affiliation.
